# Analysis of Reasonable Respiratory Efficiency in Tennis Competition and Training Environment Based on Cloud Computing

**DOI:** 10.1155/2022/4289667

**Published:** 2022-04-18

**Authors:** Honghua Ren, Wang Dan

**Affiliations:** Xijing University, Xi'an 710123, Shaanxi, China

## Abstract

Competitive tennis is developing in the direction of quantification. How to use and give full play to all positive factors, in order to attack actively and give full play to the limits of body and psychology, breathing, as the basic metabolic function of human body, also plays a vital role in tennis. This paper studies that it plays an important role in the rationality and explosiveness of sports and the psychological and physiological regulation in competition. The characteristics of tennis events determine the importance of scientific and rational breathing. Reasonable breathing during exercise is conducive to maintaining the basic stability of the internal environment, improving the training effect, and giving full play to the functional ability of the human body, so as to create excellent sports results. First, reduce respiratory resistance. Second, there are two methods to improve alveolar ventilation efficiency and pulmonary ventilation: increasing respiratory rate and increasing respiratory depth. When the inhalation volume is constant, the alveolar gas freshness rate depends on the functional residual volume in the alveolar cavity at the end of expiratory or before inhalation. The less functional the residual air, the more fresh air inhaled, and the higher the oxygen partial pressure in alveolar gas. An effective way to reduce the functional residual volume in the alveolar cavity is to exhale as deeply as possible, so as to ensure that more oxygen enters the body. Reasonable breathing methods can not only accelerate the excitation of the body, increase movement strength, reduce fatigue, and promote recovery but also play a vital role in the rational allocation of physical fitness and the improvement of sports performance. The purpose of this study is to provide a theoretical basis for scientific tennis training by analyzing the characteristics of tennis events, the form of breathing in tennis and the efficiency of reasonable breathing in tennis.

## 1. Introduction

From the physiological point of view, the human body's breathing under normal conditions does not need to deliberately go through the brain's thinking and then the brain sends out instructions to finally complete the gas exchange from the lungs. From the moment of birth, I learned to breathe when the brain is not fully developed [1]. Thus, breathing is a nonconscious rhythmic activity that does not require brain thinking. Similarly, in sports, athletes often deliberately consciously or unconsciously adjust the breathing rhythm in competitions and training [2]. In the fierce tennis competition, athletes are faced with tremendous psychological pressure and the need for oxygen consumption in the game. The breathing adjustment at this time is of vital importance to the players [3]. More and more athletes in modern tennis competitions scream, and they are more famous for their “squeaking” by Sharapova, Azarenka, Serena, and Schiavone. They are known as “the tennis soprano” [4]. The “roar” in the course of the game increases the spectacle of the game, but also potentially becomes an unconventional “weapon” to defeat the opponent [5]. In fact, on one hand, the psychological burden of cat light competition, on the other hand, it plays a physiological role in relaxing the body and mind, on the other hand, it can also interfere with opponents and distract attention [6]. This study is devoted to the study of reasonable respiratory efficiency in tennis and matches. It aims to provide a theoretical basis for athletes and coaches to train, compete and guide more scientifically and reasonably, and for the further development of tennis technology.

The study grasps the science in the tennis movement breathing, is in the present tennis training a new topic, in the foreign tennis circle, attaches great importance to the training and the research to the breathing and opens the breathing training course specially. By contrast, although qigong and taijiquan are well known in China, there is still no unified and scientific breathing method for tennis [7]. Especially in tennis, breathing is a serious exploration, and study of this problem in tennis should be given enough attention. Tennis is a sport with long duration. The average duration of each match is one and a half hours, and some matches even exceed 5 hours [8]. Athletes want to win the final game; physical fitness is a crucial factor among many influencing factors. Reasonable use of physical energy can not only reduce the physical energy consumption in the game but also improve the efficiency of the game [9]. From another point of view, the reasonable use of physical energy can also make up for the lack of physical energy to reduce the consumption of physical energy to a certain extent. Reasonable breathing adjustment can adjust the state of the body function during the interval of the game and can be effective in the case of high-intensity confrontation [10].

Nowadays, there are many tennis events, and the competition is becoming more and more intense and exciting. The key to the success of the competition is to give full play to the competitive ability of the athletes [11]. As a basic metabolic function of human body, breathing plays an important role in sports. Tennis is a combination of aerobic and anaerobic metabolism [12]. Anaerobic exercise is an exercise that effectively inhales and utilizes oxygen and generates heat by breathing during exercise. Aerobic exercise is characterized by long duration, heart rate does not exceed 85% of the maximum heart rate, can enhance aerobic endurance, consumption of excess fat, but not accumulation of fatigue, that is, accumulation of lactic acid [13]. These characteristics of aerobic metabolism are embodied in tennis, foe example, running and hitting, and swinging [14]. Anaerobic exercise is mainly intense exercise, cannot be sustained for a long time, will not consume as much heat as imagined, consumption is mainly sugar, almost no fat [15]. Anaerobic metabolism is mainly reflected in the explosive force and multiple consecutive attacks in the global competition or practice in competitive tennis. When tennis plays a good role in human endurance, explosiveness, cardiopulmonary function, tendon strength, related ligaments and soft tissues, and oxygen-carrying function of blood, it can also greatly improve human body coordination and reflect judgment [16]. Tennis is a sport that gradually improves its skills during the game, so breathing is important.

In this article, we present a cloud-based algorithm that is an algorithm for the analysis of reasonable respiratory performance in tennis matches and training environments.

In summary, our contributions are as follow:

1. )This algorithm is a new technology based on reasonable respiratory efficiency analysis in tennis competition and training environment.
2. )This technology is widely applicable in the environment of cloud computing and is highly applicable to the analysis of reasonable respiratory efficiency in most tennis matches and training environments.
3. )Higher accuracy, stronger operability, and better visualization effect.

## 2. Related Work

The correct breathing method is very important in any sports. Whether the adjustment of the breathing rhythm of the athlete in the competition is correct plays a crucial role in the outcome and the performance of the game. Emotional fluctuations are inevitable at such tight moments such as bureaus, counts, and match points [17]. In this case, the correct breathing method can stabilize the mood, improve the concentration so that you can maintain a good hitting rhythm, and increase the chance of winning this score in the game. In 2003, some scholars expressed the importance of intermittent hypoxia to improve endurance and submaximal exercise efficiency. Breathing is very important in tennis training and competition. In normal training and competition, coaches will require athletes to adjust their on-court state through the speed and depth of breathing rhythm. When a player gets the chance to kill the ball, he needs to exhale at the fastest speed; and when holding the ball or passive defense, he should use a stable posture to control the ball, and while hitting the ball, his exhalation is correspondingly slow [18].

Since the muscles that complete respiration are skeletal muscles, the rhythm of respiration can also be consciously controlled by the brain [19]. In 2003, based on the high-frequency regulation of heart rate variability during exercise in patients with COPD, some scholars believed that the heart rate of athletes would change with the change of intensity during the competition. After high-intensity exercise, the heart rate will not recover immediately. At this time, deep breathing is helpful to reduce the heart rate, reduce energy consumption, and improve the work efficiency of the cardiovascular system [20]. In 2012, some scholars believed that abdominal breathing could make hormones and autonomic nervous system function harmoniously, regulate emotions, and promote mental and physical health. Abdominal breathing itself is also used as an effective and convenient psychological skill to help athletes quickly adjust their emotions, relieve tension and restore calm in the on-the-spot scenario [21]. When the exercise intensity increases, the heart rate also increases. When the body load is stopped, the body needs to repay the oxygen deficiency generated during exercise, and the heart rate cannot immediately return to the heart rate level when it is quiet [22]. Whether it is a professional tennis player or an amateur tennis enthusiast, you should learn and master the correct breathing method in your usual game and training.

## 3. Materials and Methods

With the development of modern tennis, more and more players have adopted open footwork and Western style forehand shots. The root cause of this phenomenon is the development of modern tennis in the direction of power. According to statistics, in the high-level tennis match, the world tennis elite athletes can serve and hit the ball at a speed of 200 km/h (about 60 m/s), even if the speed of the second ball reaches 180 km/h. High ball speeds require athletes to have good explosive power, otherwise they will find it difficult to get a good ranking in the world of tennis. The explosive power of a tennis player's shot is inseparable from the coordinated power of all parts of his body. The development of modern men's tennis technology tends to be refined and comprehensive. The bottom line playing method occupies a major position in the game. In the fierce confrontation, tennis players need to change attack and defense frequently, which leads to a large number of multibeat stalemate in the game. In this case, in order to win the game, the requirements for hitting the ball not only need good stability but also require good speed of hitting the ball. Therefore, this paper takes the stability and speed of hitting the ball as the key point. Explosive force refers to the force issued in unit time, which is the embodiment of power and the main form of speed force. Tennis players usually use explosive force in the attack ball in the game or training. It is in line with the trend of tennis towards power and speed.

In sports, most people would choose the way of exhaling and inhaling gas in the fastest speed because it is believed that such fast breathing will let more oxygen into the body, so as to better alleviate the sense of urgency of breathing and improve the effect of exercise. When the ventilation volume exceeds a certain level during the exercise, the sense of breathing urgency increases with the increase of ventilation volume. But the structure of the human body determines that not all gases in gas exchange can be exchanged. Oxygen in the trachea cannot be exchanged because of the existence of physiological invalid chambers. Only part of the lungs can be exchanged. So how much ventilation in the lungs is the focus we should pay attention to. Physical strain is inevitable when the game reaches its climax or key points. In a tense state, it will lead to breathing and further aggravate the serious shortage of oxygen supply in the body. You can relax by breathing. Especially deep breathing is more effective (as shown in Table 1). Under the same ventilation volume, the amount of alveolar ventilation directly determines the degree of gas exchange. The more the alveolar ventilation, the more the gas exchange is completed.

Each sport is a combination of individual technical movements, and each movement can be divided into many individual movements. A single action is the basic element of a set of actions. Each action can be divided into two stages: preparation and completion. The “preparation” corresponds to the “breathing” stage, and the “completion” corresponds to the “call” stage. Sometimes, there is a process of “closure” in the stage of “inhalation”. The whole breathing process is a process of energy accumulation and release. In tennis technology, the action of each technical lead racket is the preparation stage, and the swing and batting follow-up is the completion stage of the action. Athletes often breathe in and hit the ball at the same time as starting the racket. The radian of the stroke is different, and the exhalation time is different. Exhale quickly if you want to swing fast. During the game, players can often hear the sound of loud exhale, which is usually emitted at the moment of hitting the ball, releasing the accumulated energy at the moment of hitting the ball, and adding this energy to the ball to generate power and increase the attacking power of the shot.

Tennis sport is characterized by a short time of high intensity and hard work and a short time of intermittent alternating long strenuous exercise. Through cloud computing, the Laplace pyramid multiresolution decomposition of the efficiency of reasonable breathing in tennis match and training environment can be obtained, and the expression of generation formula and the expression of result formula can be expressed as follows:

(1) $$\begin{array}{c} x_{ij}=x_{ij}^{T}+\text{rand}\left(0,1\right)\cdot \left(x_{ij}^{U}-x_{ij}^{L}\right),\\ p_{i}=\frac{\left(1/{\text{fit}}_{i}\right)}{\sum_{i=1}^{N_{p}}\left(1/{\text{fit}}_{i}\right)}. \end{array}$$

In order to obtain the poor performance of reasonable breathing in the tennis match and training environment, and to sample the respiratory rate during tennis, there are

(2) $$\begin{array}{c} v_{ij}=x_{ij}^{T}+\omega \cdot \Phi \cdot \left(x_{ij}^{T}-x_{rj}^{T}\right). \end{array}$$

The baseband of different spatial respiratory frequencies is obtained by spatial filtering, while time-domain filtering is a band-pass filtering for each baseband after respiratory filtering. The purpose is to obtain the respiratory efficiency signal of interest, in the form of

(3) $$\begin{array}{c} f_{i}=\sum_{i=1}^{N}\frac{\left\|y_{i}-y_{mi}\right\|}{\left\|y_{i}-{\bar{y}}_{i}\right\|}. \end{array}$$

Normal breathing cycle is within a range, different people at different times will have different breathing cycle, and the system real-time self-renewal of the current normal breathing average is as follows:

(4) $$\begin{array}{c} {\ddot{r}}_{sa}=-k\rho_{SR}C_{R}\frac{A}{m}{\hat{r}}_{s}. \end{array}$$

The formula for the *r*th breath is as follows:

(5) $$\begin{array}{c} {\ddot{r}}_{\text{da}}=-\frac{1}{2}C_{D}\frac{A}{m}\rho \dot{r}\dot{r}. \end{array}$$

Then the formula for judging reasonable breathing in general tennis competition and training environment is as follows:

(6) $$\begin{array}{c} x_{\left(i+1\right)j}^{\text{New}}=x_{ij}+R\left(2y_{\left(i+1\right)j}-1\right). \end{array}$$

**Figure 1:**
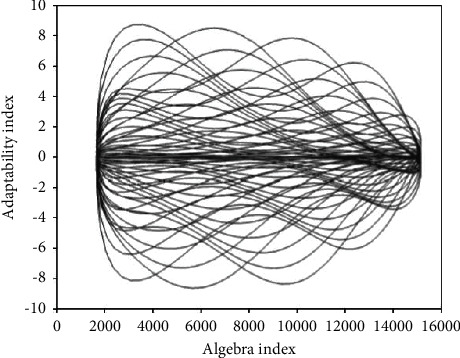
Rational respiratory efficiency fitness curve in tennis competition and training environment.

**Figure 2:**
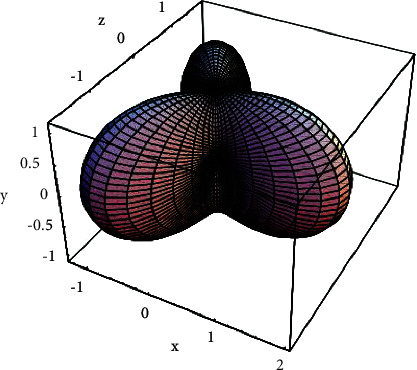
Phase space model of tennis movement breathing mapped by cloud computing.

With classification accuracy as fitness, the curve of reasonable respiratory fitness between tennis match and training environment is shown in Figure 1. When cloud computing is mapped to the phase space model of tennis breathing (Figure 2), the dimension of the parameter vector corresponds to the dimension of the phase space. The position of the end point of the vector in the phase space represents the comprehensive load of breathing in tennis matches and training, and reflects the current state of breathing efficiency. Then, the load situation and the current state of breathing efficiency are expressed in formula (6), respectively.

(7) $$\begin{array}{c} \Delta X=\Phi \Delta X_{0}+S\Delta P,\\ \frac{{\text{d}}^{2}}{\text{d}t^{2}}\left(\frac{\partial r}{\partial p}\right)=\frac{\partial \ddot{r}}{\partial r}\left(\frac{\partial r}{\partial p}\right)+\frac{\partial \ddot{r}}{\partial \dot{r}}\frac{\text{d}}{\text{d}t}\left(\frac{\partial r}{\partial p}\right)+\frac{\partial \ddot{r}}{\partial p}. \end{array}$$

Assuming that the respiratory efficiency parameter generated in the tennis competition and training environment is *P*, its point set in the cloud computing phase space is as follows:

(8) $$\begin{array}{c} \left(\begin{array}{c} \Delta X_{0}\\ \Delta P \end{array}\right)={\left({\left(\Phi S\right)}^{T}\left(\Phi S\right)\right)}^{-1}{\left(\Phi S\right)}^{T}\Delta X. \end{array}$$

The peak time difference between the statistical game and the preattraction during the training, and the waveform composed of points represents the current breathing cycle. When the period is in the normal range, the earliest value in the periodic list is deleted, and the current period is added to the linked list, and the current period is represented by the average of the linked list, and the formula is

(9) $$\begin{array}{c} M\frac{\text{d}y^{2}m}{\text{d}t^{2}}=P_{y}-Mg,\\ {\text{Dur}}_{i}=\alpha \left(et_{k}^{IA}-st_{k}^{IA}\right),\forall at,rt\in T. \end{array}$$

The basic principle of reasonable coordination between breathing and tennis is that “breathing” is the process of hair and release, and “breathing” is the process of collecting and accumulating strength. Breathing allows fresh air to fully exchange gas with blood and alveoli in the lungs, inhale oxygen, exhale carbon dioxide and other gases, and maintain the metabolic needs of the human body. Normally, oxygen uptake and carbon dioxide emissions require ventilation to be compatible with blood flow. In a quiet state, the maintenance of the adaptive relationship among ventilation, oxygen intake and carbon dioxide output is regulated by the respiratory center driving the peripheral respiratory movement, or by the cerebral cortex causing respiratory sensation, which is transmitted through the nervous system to the respiratory muscles to control breathing reasonably. In sports, some internal organs are inert to their functions, and oxygen intake cannot meet the oxygen demand immediately. At this time, breathing plays a role in regulating, controlling, increasing lung ventilation, reducing oxygen debt, and enhancing metabolic capacity, so as to facilitate exercise effects and performance. In tennis competitions, breathing has its own special laws, methods, and functions.

## 4. Results

Breathing itself is a kind of movement; it is through the contraction and relaxation of breathing muscles to complete the whole breathing movement. Respiratory muscle contraction is a powerful assistant in many cases when it causes the expansion of the thorax (upper and lower, front and back, left and right directions), and the relaxation of the respiratory muscle makes the thorax retract. Take the open forehand stroke of the right hand as an example. When the clapper leads backward, the center of gravity drops, the feet are parallel to the baseline, the hip rotates, and the right hand clapper rotates toward the waist of the back lead belt. At this time, both arms are in the abduction position, the thoracic opening stage of inspiration, diaphragm, and intercostal muscle contraction, while the right pectoralis major muscle in tension, the right trapezius muscle, and latissimus dorsi muscle in contraction. The rectus abdominis is in the state of stretching to the upper right, which seems to be a simple racket action, and most of the respiratory muscles have been transferred into the force program of the action. In the short lead has completed a process of preparation, let the active force of the muscles to fully relax, for the impact of the shot to make full preparation. When you hit the ball, kick your legs and turn your waist, drive the upper body to rotate, hold the racket and quickly wave your right hand to hit the ball. At this point, the left arm is in a natural state in front of the body, the right arm with the adduction, hip rotation to the left. In the process of hitting the ball, the intercostal muscles and diaphragm are actively relaxed, the trapezius dorsi is stretched, the pectoralis major is contracted, and the rectus abdominis is contracted to expel the body gas from the body. The rectus abdominis stretches slightly as it swings to the left of the hip, so there is often a “return of breath” after the stroke is completed.

Tennis is a movement that alternates with aerobic and anaerobic metabolic movements but is dominated by aerobic metabolism. The interval of exercise time in tennis matches is mainly aerobic metabolism, and high-level tennis players must have good aerobic capacity (Table 2). As can be seen from the table below, the difference in maximum oxygen uptake between elite tennis players and ordinary tennis players is significant. The higher the level of tennis players, the better the endurance quality. The maximum oxygen uptake is an important index to measure the aerobic metabolism ability. The higher the maximum oxygen uptake is, the higher the aerobic metabolism ability of tennis players will be. Improving aerobic metabolism can help athletes to improve their ability to react quickly. Moderate breathing during competition is important for improving endurance on the field.

**Figure 3:**
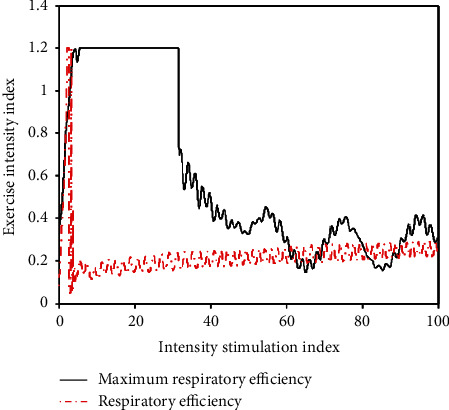
Ventilator body skill level.

The research shows that the body's respiratory efficiency is relatively low at the beginning of tennis with low intensity load. After a slight load stimulation, the respiratory efficiency rises rapidly. After a period of fixed intensity stimulation, it will remain in a relatively stable range. During this period, if the intensity remains unchanged, the respiration remains basically stable. If the intensity of exercise continues to increase, the body must mobilize more energy substances in order to adapt to the greater energy consumption. At this time, the number of breaths will increase, and the respiratory efficiency will also increase. When the exercise intensity reaches the maximum value of the body, the respiratory performance will also reach a maximum. The maximum respiratory performance as an indicator can reflect the level of the body's function to a certain extent and can also control and understand the exercise intensity through breathing (Figure 3). According to the different reactions of the body, the changes in respiratory performance in tennis were divided into five grades (Table 3).

**Figure 4:**
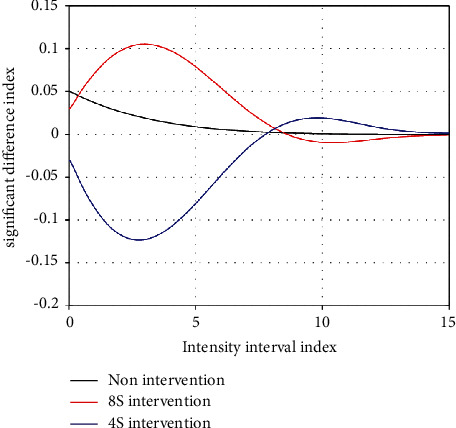
Significant difference test for respiratory performance in different intensity intervals.

**Figure 5:**
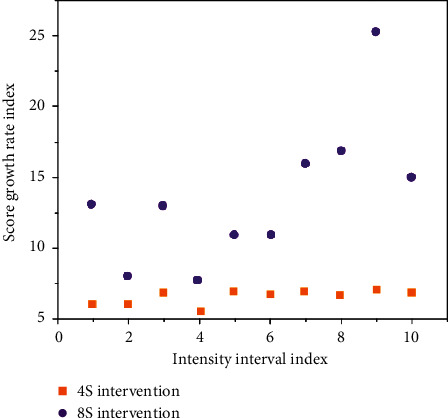
Growth rate line chart of the growth rate of different exercise intensity intervals in the intervention group compared with the nonintervention group.

The subjects were asked to collect data three times, each time including five different heart rate intensity intervals (<103, 104–115, 116–120, 121–125, and 126–130). The first data acquisition was conducted without any intervention. The second one was to learn how to breathe deeply and then to adjust the breath for 4 seconds during the 20-second interval during the test. Meanwhile, the score and ball speed of the test were recorded. For the third time, 8 seconds of deep breathing adjustment were used during the test, while scores and ball speed were recorded. Each subject was tested three times, once a week, for a total of three weeks. The experimental results are shown in Table 4 and Figure 4, and the growth rate of the experimental scores is shown in Table 5 and Figure 5.

**Figure 6:**
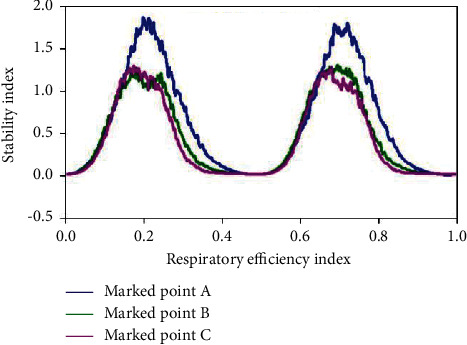
Effect of breathing on the stability of teeing.

**Figure 7:**
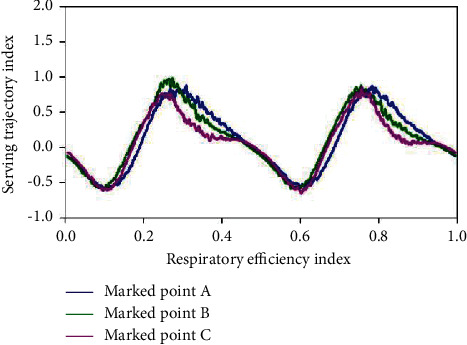
Effect of breathing on the stability of teeing.

Of all the skills in tennis, serve is the only one that can be accomplished by itself almost without the influence of an opponent. The aggressiveness of service includes the ball's landing point, speed, strength, rotation, and arc. The aggressiveness of the ball is based on stability, which is divided into throwing stability and hitting stability. The factors affecting stability include many aspects, among which the effect of reasonable breathing on stability is very important. Figures 6 and 7 show the effect of breathing on serve stability. The power transmission lines of serve are legs, hips, shoulders, and arms. In this process, with the completion of the action, when hitting the ball, the core area of the body needs to be in a stable state to increase the stability of the ball. Holding your breath for a short period of time puts your nervous system in a state of tension that increases the instant power of your shot.

Reasonable breathing during movement can achieve the effect of adjusting the pace and frequency by controlling the depth and frequency of breathing. It can provide more powerful muscle traction and make the movement faster. The movement can also control or regulate thoracic undulation effectively using suffocation reasonably, coordinate the core area muscle group, adjust the abdominal muscle group, muscle in a tense state, make the swing arm move more quickly, make the core area more stable, make the swing limbs get a relatively stable fulcrum, and make the movement more stable. Tennis technology includes bottom line technology and net front technology, bottom-line technology refers to the forehand and backhand technology, net front technology refers to the volley and near net high-pressure technology. In the process of hitting the ball, breathing can promote the dynamic stereotyping of the action, increase the hitting power and improve the hitting stability.

## 5. Conclusion

Modern tennis players have fine techniques and tactical changes. When two athletes with comparable levels of competition play on the court, the details of the advantage determine the final success or failure. Therefore, the role of breathing in tennis matches is highlighted. The scientific breathing method plays a very positive role in regulating the physical and psychological state of the athletes on the field. It helps the athletes to play stably and helps the physical fitness to maintain and help control the rhythm on the field. This will not only increase the athlete's momentum on the field but also promote the athletes to show the best level of competition in the game. Therefore, it is suggested that both basic tennis training and professional tennis training should pay attention to the training of athletes' scientific breathing methods. In the course of tennis training and competition, we should pay attention to the close cooperation of reasonable breathing and movement and batting movements, and adjust breathing so as to make the breathing rhythm, depth, form and movement structure, rhythm, power sequence, and strength closely coordinate and coordinate, so as to form a scientific, systematic, and stable action efficiency.

## Figures and Tables

**Table 1:** Comparison of pulmonary ventilation and tidal volume at different respiratory frequencies.

Respiratory frequency (times/min)	Tidal volume (mL)	Pulmonary ventilation volume (mL/min)	Alveolar ventilation volume (mL/min)
8	1050	7600	6750
16	550	7600	5560
32	250	7600	3420

**Table 2:** Maximum oxygen uptake of tennis players.

Excellent		Ordinary		Significance test
Average value	Standard deviation	Average value	Standard deviation	
55.8	7.66	61.37	6.51	*P* < 0.05

**Table 3:** Respiratory efficacy comparison table.

	First interval	Second interval	Third interval	Fourth interval	Fifth interval
Maximum percentage	45%–55%	56%–65%	66%–71%	72%–79%	80%–90%
Respiratory rate	<103	104–115	116–120	121–125	126–130

**Table 4:** Significant difference test of respiratory performance in different intensity intervals.

	<103	104–115	116–120	121–125	126–130
No intervention	61.1	63.4	66.5	56.4	48.2
4 s/times	62.4	67.2	71.3	65.1	54.6
8 s/times	57.5	63.8	71.7	70.8	59.6

**Table 5:** Growth rate of serve scores in different exercise intensity intervals of intervention group versus nonintervention group.

	<103	104–115 (%)	116–120 (%)	121–125 (%)	126v130 (%)
4 s/times	1.23%	6.67	3.41	20.67	18.47
8 s/times	−5.69%	6.13	5.31	25.54	33.29

## Data Availability

The data used to support the findings of this study are included within the article.

## References

[1] Klaassen F. J. G. M., Magnus J. R. (2009). The efficiency of top agents: an analysis through service strategy in tennis. *Journal of Econometrics*.

[2] Martin C., Bideau B., Ropars M., Delamarche P., Kulpa R. (2014). Upper limb joint kinetic analysis during tennis serve: a. *Scandinavian Journal of Medicine & Science in Sports*.

[3] Baba R., Nagashima M., Goto M. (1996). Oxygen intake efficiency slope: a new index of cardiorespiratory functional reserve derived from the relationship between oxygen consumption and minute ventilation during incremental exercise. *Nagoya Journal of Medical Science*.

[4] Fairbarn M., Coutts K., Pardy R., McKenzie D. (1991). Improved respiratory muscle endurance of highly trained cyclists and the effects on maximal exercise performance. *International Journal of Sports Medicine*.

[5] Reybrouck T. (2001). Cardiorespiratory exercise function after the arterial switch operation for transposition of the great arteries. *European Heart Journal*.

[6] Samaras D., Pison C., Cano N., Lejeune H., Roth H., Pichard C. (2013). Pp133-Sun adequate selection of chronic respiratory failure malnourished patients may improve clinical outcomes and efficiency of a multimodal intervention: a p analysis of a p. *Clinical Nutrition*.

[7] Chase P. J., Kenjale A., Cahalin L. P. (2013). Effects of respiratory exchange ratio on the prognostic value of peak oxygen consumption and ventilatory efficiency in patients with systolic heart failure. *Journal of the American College of Cardiology: Heart Failure*.

[8] Keegan J., Jhooti P., Babu‐Narayan S. V., Drivas P., Ernst S., Firmin D. N. (2014). Improved respiratory efficiency of 3D late gadolinium enhancement imaging using the continuously adaptive windowing strategy (CLAWS). *Magnetic Resonance in Medicine*.

[9] Baba R. (2000). The oxygen uptake efficiency slope and its value in the assessment of cardiorespiratory functional reserve. *Congestive Heart Failure*.

[10] Guazzi M., Vicenzi M., Arena R. (2012). Phosphodiesterase 5 inhibition with sildenafil reverses exercise oscillatory breathing in chronic heart failure: a long-term cardiopulmonary exercise testing placebo-controlled study. *European Journal of Heart Failure*.

[11] Kang J.-i., Jeong D.-K., Choi H. (2016). The effects of breathing exercise types on respiratory muscle activity and body function in patients with mild chronic obstructive pulmonary disease. *Journal of Physical Therapy Science*.

[12] Olson T. P., Snyder E. M., Johnson B. D. (2006). Exercise-disordered breathing in chronic heart failure. *Exercise and Sport Sciences Reviews*.

[13] Baraniuk J. N., Merck S. J. (2008). Nasal reflexes: i. *Current Allergy and Asthma Reports*.

[14] Kiers A., van der Mark T. W., Folkerts F. J., Blankena H. J., Woldring M. G., Peset R. (1982). An electromagnetic valve for rebreathing exercise studies. *Journal of Applied Physiology*.

[15] Seo K., Hwan P. S., Park K. (2017). The effects of inspiratory diaphragm breathing exercise and expiratory pursed-lip breathing exercise on chronic stroke patients’ respiratory muscle activation. *Journal of Physical Therapy Science*.

[16] Yong M.-S., Lee H.-Y., Lee Y.-S. (2017). Effects of diaphragm breathing exercise and feedback breathing exercise on pulmonary function in healthy adults. *Journal of Physical Therapy Science*.

[17] Tsunashima Y., Vedam S., Dong L. (2008). Efficiency of respiratory-gated delivery of synchrotron-based pulsed proton irradiation. *Physics in Medicine and Biology*.

[18] Katayama K., Matsuo H., Ishida K., Mori S., Miyamura M. (2003). Intermittent hypoxia improves endurance performance and submaximal exercise efficiency. *High Altitude Medicine & Biology*.

[19] Jacobs R. A., Siebenmann C., Hug M., Toigo M., Meinild A. K., Lundby C. (2012). Twenty‐eight days at 3454‐m altitude diminishes respiratory capacity but enhances efficiency in human skeletal muscle mitochondria. *The FASEB Journal*.

[20] Bartels M. N., Jelic S., Ngai P., Basner R. C., DeMeersman R. E. (2003). High-frequency modulation of heart rate variability during exercise in patients with COPD. *Chest*.

[21] Forster H. V., Haouzi P., Dempsey J. A. (2012). Control of breathing during exercise. *Comprehensive Physiology*.

[22] Arioli S., Zambelli D., Guglielmetti S. (2013). Increasing the h-dependent respiratory efficiency of l by inhibition of lactate dehydrogenase. *Applied and Environmental Microbiology*.

